# RETRACTION: Value of Circular RNA 0007385 in Disease Monitoring and Prognosis Estimation in Non–Small‐Cell Lung Cancer Patients

**DOI:** 10.1002/jcla.70242

**Published:** 2026-04-13

## Abstract

**RETRACTION**: Y. Lin, W. Su, G. Lan, “Value of Circular RNA 0007385 in Disease Monitoring and Prognosis Estimation in Non–Small‐Cell Lung Cancer Patients,” *Journal of Clinical and Laboratory Analysis* 34, no. 8 (2020): e23338, https://doi.org/10.1002/jcla.23338.

The above article, published online on 14 July 2020 in Wiley Online Library (wileyonlinelibrary.com), has been retracted by agreement between the journal Editor‐in‐Chief, Rong Fu; and Wiley Periodicals, LLC. The retraction has been agreed due to concerns raised by a third party, which identified compromised data and systematic issues in the published article. The authors and their institutes were unresponsive when these concerns were brought to their attention. Accordingly, the article is retracted as the editors have lost trust in the reliability of the data and the results presented.

**RETRACTION**: Y. Lin, W. Su, G. Lan, “Value of Circular RNA 0007385 in Disease Monitoring and Prognosis Estimation in Non–Small‐Cell Lung Cancer Patients,” *Journal of Clinical and Laboratory Analysis* 34, no. 8 (2020): e23338, https://doi.org/10.1002/jcla.23338.

The above article, published online on 14 July 2020 in Wiley Online Library (wileyonlinelibrary.com), has been retracted by agreement between the journal Editor‐in‐Chief, Rong Fu; and Wiley Periodicals, LLC. The retraction has been agreed due to concerns raised by a third party, which identified compromised data and systematic issues in the published article. The authors and their institutes were unresponsive when these concerns were brought to their attention. Accordingly, the article is retracted as the editors have lost trust in the reliability of the data and the results presented.

